# Mechanism of Nucleic Acid Unwinding by SARS-CoV Helicase

**DOI:** 10.1371/journal.pone.0036521

**Published:** 2012-05-15

**Authors:** Adeyemi O. Adedeji, Bruno Marchand, Aartjan J. W. te Velthuis, Eric J. Snijder, Susan Weiss, Robert L. Eoff, Kamalendra Singh, Stefan G. Sarafianos

**Affiliations:** 1 Christopher Bond Life Sciences Center, Department of Molecular Microbiology and Immunology, University of Missouri, School of Medicine, Columbia, Missouri, United States of America; 2 Molecular Virology Laboratory, Department of Medical Microbiology, Center of Infectious Diseases, Leiden University Medical Center, Leiden, The Netherlands; 3 Department of Microbiology, University of Pennsylvania, School of Medicine, Philadelphia, Pennsylvania, United States of America; 4 Department of Biochemistry and Molecular Biology, University of Arkansas for Medical Sciences, Little Rock, Arkansas, United States of America; Institut National de la Santé et de la Recherche Médicale, France

## Abstract

The non-structural protein 13 (nsp13) of Severe Acute Respiratory Syndrome Coronavirus (SARS-CoV) is a helicase that separates double-stranded RNA (dsRNA) or DNA (dsDNA) with a 5′→3′ polarity, using the energy of nucleotide hydrolysis. We determined the minimal mechanism of helicase function by nsp13. We showed a clear unwinding lag with increasing length of the double-stranded region of the nucleic acid, suggesting the presence of intermediates in the unwinding process. To elucidate the nature of the intermediates we carried out transient kinetic analysis of the nsp13 helicase activity. We demonstrated that the enzyme unwinds nucleic acid in discrete steps of 9.3 base-pairs (bp) each, with a catalytic rate of 30 steps per second. Therefore the net unwinding rate is ∼280 base-pairs per second. We also showed that nsp12, the SARS-CoV RNA-dependent RNA polymerase (RdRp), enhances (2-fold) the catalytic efficiency of nsp13 by increasing the step size of nucleic acid (RNA/RNA or DNA/DNA) unwinding. This effect is specific for SARS-CoV nsp12, as no change in nsp13 activity was observed when foot-and-mouth-disease virus RdRp was used in place of nsp12. Our data provide experimental evidence that nsp13 and nsp12 can function in a concerted manner to improve the efficiency of viral replication and enhance our understanding of nsp13 function during SARS-CoV RNA synthesis.

## Introduction

Severe Acute Respiratory Syndrome Coronavirus (SARS-CoV) is the causative agent of SARS, a life-threatening illness characterized by flu-like syndrome such as high fever, pneumonia, dyspnoea, infiltrates on chest radiography and sometimes respiratory failure [1]. The global spread of SARS caused hundreds of deaths and had enormous negative impact to the global economy in 2003–2004. SARS-CoV is a positive single-stranded (ss) RNA virus with one of the largest known RNA genomes (∼29.7 kb) [2], [3]. SARS-CoV gene expression involves poorly understood transcriptional and translational regulatory mechanisms. Following infection, there is translation of two large replicative polyproteins, the pp1a (the ORF1a polyprotein) and the pp1ab (the polyprotein made from the ORF1a and ORF1b through a −1 ribosomal frameshift during translation). These polyproteins are processed by the virus-encoded papain-like proteinase (PL^pro^; residing in nsp3) and nsp5, a 3C-like proteinase [4], [5], [6], [7], [8], [9], [10]. This auto-proteolysis leads to formation of 16 non-structural proteins, including an RNA-dependent RNA polymerase (RdRp) and an NTPase/helicase that are known as non-structural proteins 12 and 13, respectively (nsp12 and nsp13). These are likely to form the core of membrane-bound replication-transcription complexes in double-membrane vesicles at perinuclear regions [11]. Helicases are motor proteins that unwind double-stranded nucleic acids into two single-stranded nucleic acids by utilizing the energy derived from nucleotide hydrolysis [12], [13], [14], [15], [16], [17], [18], [19]. Although, helicases were initially thought as molecular engines that unwind nucleic acids during replication, recombination, and DNA repair [16], recent reports have shown that they are also involved in other biological processes, including displacement of proteins from nucleic acid, movement of Holliday junctions, chromatin remodeling, catalysis of nucleic acid conformational changes [20], [21], [22], [23], [24], [25], several aspects of RNA metabolism, including transcription, mRNA splicing, mRNA export, translation, RNA stability and mitochondrial gene expression [11]. Defects in helicase function have been associated with some human diseases, including Bloom's syndrome, Werner's syndrome, and Xeroderma Pigmentosum [26], [27], [28], [29]. The specific function of nsp13 in SARS-CoV replication has yet to be established. It is thought to interact with other SARS-CoV non-structural proteins, such as nsp7, nsp8 and nsp12 [30], [31], [32] and probably contribute to the formation of a replication complex, but the effect of these interactions on nsp13 unwinding activity is unknown.

To understand the mechanism of nucleic acid unwinding and/or translocation by nsp13 we need to determine the kinetic parameters associated with the reaction mechanism, such as the unwinding rate, unwinding processivity, directional bias, step size and stoichiometry of ATP coupling [33]. Previously, basic biochemical characterization of nsp13 demonstrated that nsp13 can unwind both double-stranded DNA and RNA in a 5′-3′ direction, and it can hydrolyze all deoxyribonucleotide and ribonucleotide triphosphates [34], [35]. These studies were conducted using constructs of nsp13 that were fused with maltose binding protein (MBP-nsp13) and hexahistidine (H_6_-nsp13). Additional studies demonstrated that the amplitude of DNA unwinding by H_6_-nsp13 increases with longer 5′-overhang containing DNA substrates [36]. These data advanced our understanding of nsp13 function [37], [38], but they do not address key mechanistic details such as the kinetic step size, rate of nucleic acid unwinding, and the ATP-coupling stoichiometry. Here, we present a minimal kinetic mechanism of nsp13.

Because nsp13 has 26 cysteine residues, 14 of which are highly conserved [34] and likely (at least for some of them) to participate in disulfide bonds, we hypothesized that if we expressed the protein in a eukaryotic expression system it would be better folded and more active than that expressed in bacteria. Hence, we prepared GST-nsp13 using a baculovirus expression system and this construct was primarily used to characterize in detail the kinetics of nucleic acid unwinding by SARS-CoV helicase, but we also carried out similar analyses with MBP-nsp13 and H_6_-nsp13 and compared them with GST-nsp13 to ensure that we were not measuring a GST-related artifact. We found that GST-nsp13 was significantly more active than the other variants and had activity comparable to other helicases [39]. In our enzyme characterization, we used both RNA and DNA substrates, as the biological role of SARS-CoV nsp13 is expected to be unwinding of RNA substrate. Moreover, we determined the effect of nsp12 on the mechanism of nsp13. Our data demonstrate that regardless of the type of fusion tag or substrate use, the helicase activity of nsp13 is enhanced in the presence of nsp12, suggesting that these proteins also interact in a functional replication complex and that their interaction contributes to the efficiency of viral replication. Our results allow mechanistic comparisons with other viral or eukaryotic helicases and set the stage for studying the intriguing evolutionary relations between helicases from nidoviruses and other groups of positive sense RNA viruses.

## Results

To monitor nucleic acid unwinding we used a standard ‘all or none’ helicase assay, which directly monitors the final product of the reaction (ssDNA or ssRNA). This assay has been successfully used to study the mechanism of several helicases and decipher the intermediates and kinetic parameters of the reaction [37], [38], [40], [41], [42], [43].

### Comparison of GST-nsp13, MBP-nsp13, and H_6_-nsp13 helicase and ATPase activities

**Figure 1:**
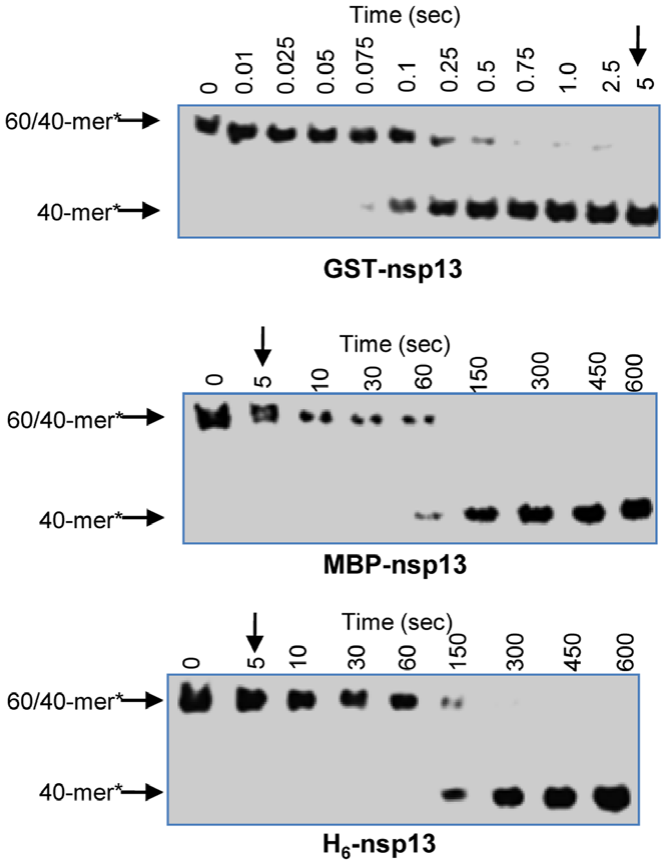
Comparison of unwinding activity of three nsp13 variants. Comparison of the helicase activity at varying time points for GST-nsp13 (Glutathione S-Transferase-tagged nsp13), MBP-nsp13 (Maltose Binding Protein-tagged nsp13), and H_6_-nsp13 (hexahistidine-tagged nsp13) using 100 nM of each enzyme and 5 nM 60/40-mer (30ss:30ds) as substrate at 30°C. The products were separated and analyzed by 6% non-denaturing PAGE. Time points for the GST-nsp13-catalyzed unwinding experiments are more than 100 times smaller than for the MBP-nsp13- and H_6_-nsp13-catalyzed experiments (0.01–5 *vs.* 5–600 seconds).

**Figure 2:**
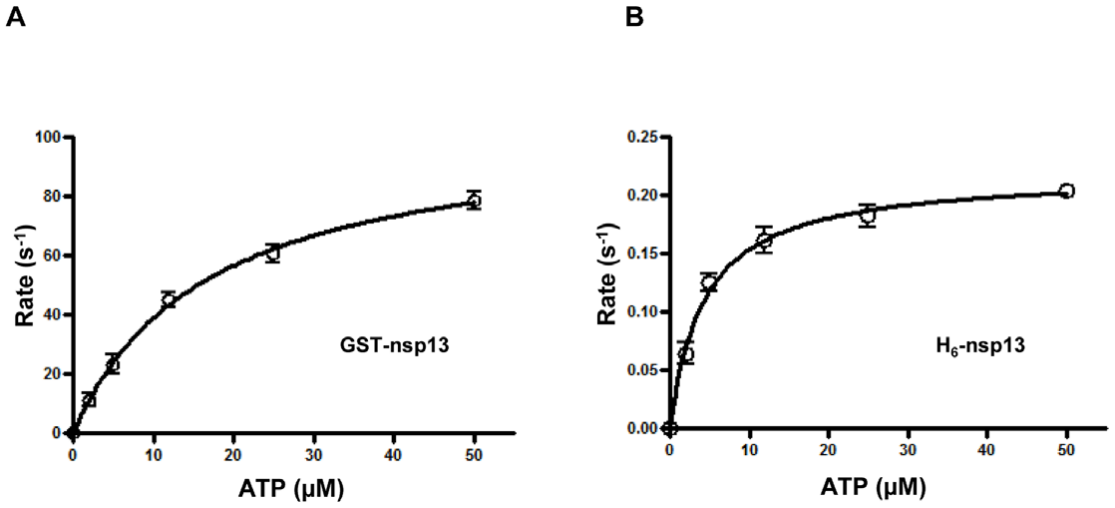
Hydrolysis of ATP during unwinding of dsDNA by GST-nsp13 and H_6_-nsp13. GST-nsp13 or H_6_-nsp13 was pre-incubated with 31/18-mer and rapidly mixed with various concentrations of γ^-32^P-ATP (2 µM, 5 µM, 12 µM, 25 µM and 50 µM,) for various reaction times (0.005–0.5 s for GST nsp13 and 0.005–5 s for H_6_-nsp13). The reaction products were separated by thin-layer chromatography and visualized by autoradiography. ATP hydrolysis was quantitated as amount of Pi released and plotted against reaction time. The data were fit to a single exponential equation to calculate initial hydrolysis rates. The derived rates were plotted against ATP concentration and the data points were fit to a hyperbolic function to determine the (A) GST-nsp13 optimal ATP hydrolysis rate *k_hydro(ATP)_* 104.1±4. s^−1^ and (B) H_6_-nsp13 optimal ATP hydrolysis rate *k_hydro(ATP)_* 0.2±0.006 s^−1^ (two independent experiments).

First, we compared the unwinding activities of H_6_-nsp13, GST-nsp13 and MBP-nsp13 using 100 nM of each enzyme and 5 nM 60/40-mer (20ss:40ds) as DNA substrate. The results presented in Figure 1 show that GST-nsp13 unwound half of the substrate in ∼0.1 seconds. However, MBP-nsp13 and H_6_-nsp13 reached the same unwinding level at approximately 60 and 100 seconds, respectively. GST-nsp13 unwound the full amount of the nucleic acid in approximately 5 seconds, whereas it took more than 600 seconds for MBP-nsp13 and H_6_-nsp13 to achieve the same task. These results demonstrated that H_6_-nsp13 was the slowest of the three enzymes and that GST-nsp13 is faster than the other variants by up to three orders of magnitude. We also compared the ATPase activities of these three variants of nsp13. We determined ATP hydrolysis by measuring released Pi using thin-layer chromatography and obtained the initial rate constants by fitting the data to single exponential curves. The initial rate constants were then plotted against [ATP] and the data were fit to a hyperbolic function, yielding *k_hydro(ATP)_* of 104.1±4.0 s^−1^ for GST-nsp13 and 0.22±0.006 s^−1^ for H_6_-nsp13 (Figure 2A and 2B). The MBP-nsp13 variant also showed similar rate of ATP hydrolysis as the H_6_-nsp13 (data not shown). Hence, most subsequent experiments for biochemical characterization were performed with GST-nsp13, and were generally repeated with H_6_-nsp13 and MBP-nsp13.

### Comparison of dsDNA and dsRNA Unwinding by nsp13

**Figure 3:**
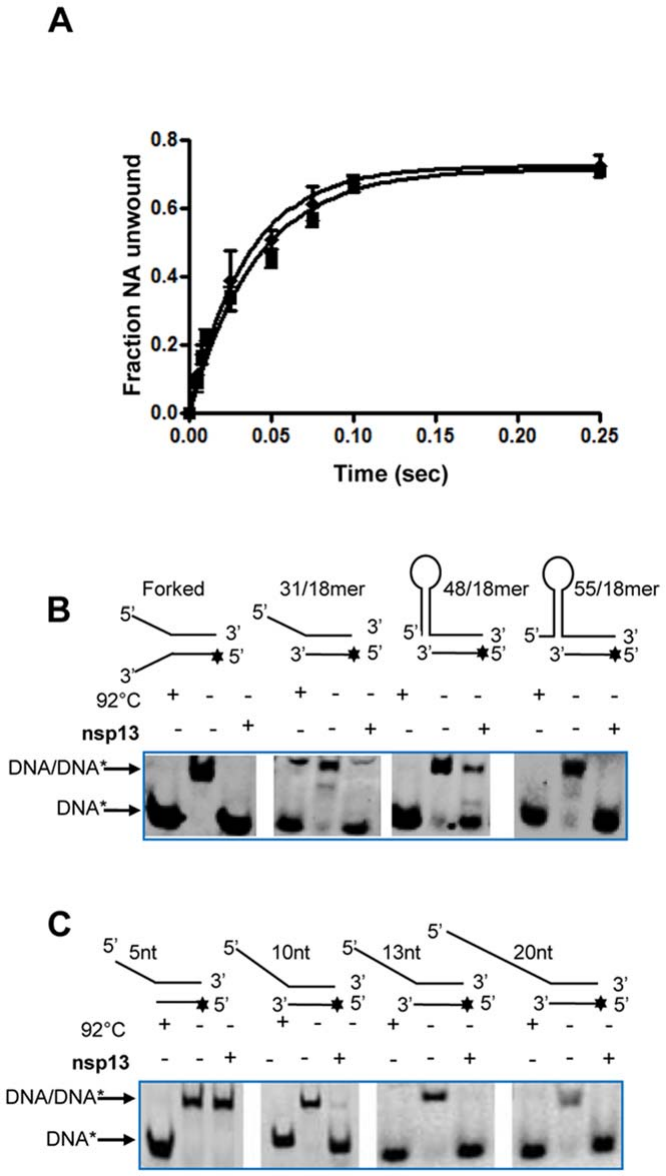
Substrate specificity of nsp13. (A) Comparison of helicase activity of GST-nsp13 using same sequence of dsDNA (♦) or dsRNA (▪) 38/18-mer substrates under conditions described in the methods section. B) Four different substrates were designed as shown in Supplementary Fig. 1 (forked substrate, 31/18-mer, 48/18-mer, and 55/18-mer). The helicase activity of 100 nM nsp13 was measured with 5 nM of each of the substrates at 30°C for 5 secs and the products were separated on a non-denaturing 6% polyacrylamide gel and visualized using a PhosphorImager. C) Four different substrates with 5′ overhang lengths varying from 5 to 20 nucleotides were designed to determine the minimum length of loading strand required by nsp13 to efficiently unwind its substrate. The helicase activity of nsp13 (10 nM) was assessed on these substrates (5 nM each) at 30°C for 10 mins and the products were separated on a non-denaturing 6% polyacrylamide gel and visualized using the PhosphorImager.

Previous studies have shown that some helicases exhibit a clear preference for either RNA or DNA [11], [44], [45], [46], [47]. To determine if GST-nsp13 prefers one substrate over the other, we designed 38/18-mer DNA and RNA duplexes (Figure S1) of the same sequence and monitored their unwinding rates under single-turnover conditions. The time course of DNA and RNA unwinding in Figure 3A demonstrates that GST-nsp13 does not exhibit a significant preference for either RNA or DNA substrates. A similar lack of preference was also observed for MBP-nsp13 and H_6_-nsp13 (data not shown) and was consistent with previous reports for these two enzymes [34], [35]. Henceforth, most experimental analysis was carried out using DNA substrates.

### Substrate Structure Specificity

Similar to previous reports for the MBP-nsp13 and H_6_-nsp13 variants [6], [34], [35] we also found that GST-nsp13 unwinds DNA and RNA substrates containing either a ‘fork’ or a 5′-end single-stranded overhang (Figure 3B and Figure S1). None of the nsp13 variants could unwind substrates with 3′-end single-strand overhang or blunt-end DNA and RNA duplexes [6], [34], [35]. However, we show here that nsp13 can unwind nucleic acid secondary structure elements (a stem-loop structure). This was shown using the 48/18-mer and 55/18-mer substrates (Figure S1), which mimic one of the stem loop structures present at the 5′ UTR of SARS-CoV genome. These two substrates (48/18-mer and 55/18-mer) differed in the length of 5′ single strand overhang before the loop (Figure 3B and Figure S1). The 55/18-mer substrate was readily unwound in contrast to the unwinding of 48/18-mer, which was only partially unwound suggesting that nsp13 can unwind stem loops provided the substrate has a 5′-end overhang loading region (Figure 3B).

### Minimum Overhang Length Requirement

To investigate whether nucleic acid unwinding depends on the loading strand length and to determine the minimum length of 5′-single-stranded (ss) overhang required for efficient helicase activity we prepared 4 partial duplex DNA substrates (Figure S1 and Figure 3C) with varying lengths of 5′-end ssDNAs (between 5- to 20-bases), but contained same length of dsDNA duplex region (23/18-mer, 28/18-mer, 31/18-mer, and 38/18-mer). Using these substrates we monitored the unwinding activity of GST-nsp13. GST-nsp13 could not unwind the 23/18-mer that has only 5 bases ssDNA at the 5′-end, but it did unwind the 28/18-mer (10 bases ssDNA at 5′-end), 31/18-mer (13 bases ssDNA at 5′-end), and 38/18-mer (20 bases at 5′-end), suggesting that it requires more than 5-base-long 5′-loading strand (Figure 3C).

### Measurement of the Kinetic Step Size for GST-nsp13

**Figure 4:**
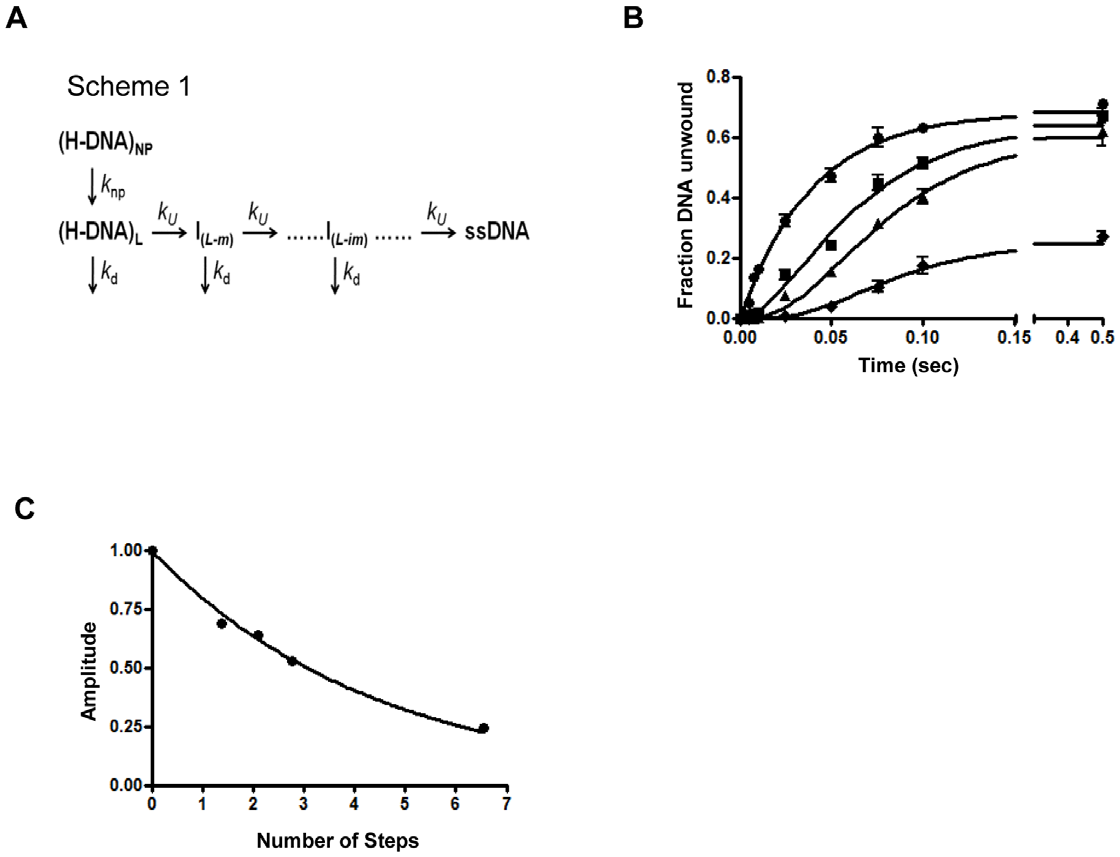
GST-nsp13-catalyzed unwinding of dsDNA of varying lengths, performed under single-turnover conditions. (A) The minimal kinetic mechanism of nucleic acid unwinding by nsp13. The minimal reaction scheme describing a series of n sequential steps of helicase-catalyzed translocation and unwinding. H-DNA, Helicase-DNA complex; I, intermediate; *k*_np_ is a macroscopic rate constant for conversion of nonproductive (H-DNA)_NP_ to productive Helicase-DNA complexes (H-DNA)_L_. Each identical step in the reaction pathway is defined by a forward rate constant, *k_u_*, and a dissociation rate constant, *k_d_*. (B) Time course of nsp13-catalyzed unwinding of dsDNA. The double stranded component of the substrates was: 18 bp (•), 30 bp (▪), 40 bp (▴), 60 bp (♦). Experiments were repeated at least twice for each substrate. (C) Plot of the amplitude of nucleic acid unwinding as a function of the number of kinetic steps determined for each substrate (for substrates with dsDNA component of 18, 30, 40, and 60 bp). Data were fit to the equation *A* = *P^n^* and the processivity, *P*, of nsp13 was estimated from the amplitude *A* and number of steps *n* and found to be 0.80±0.03.

In order to estimate the kinetic step size of the SARS-CoV helicase we performed single-turnover assays by pre-incubating excess of GST-nsp13 with partial-duplex substrates. The change in the fraction of unwound ssDNA product with time is shown in Figure 4B. The experimental data for all the substrates were fit globally to equation (1) derived from Scheme 1 (Figure 4A). The resulting fits are shown in Figure 4B and the kinetic parameters are listed in Table 1. Notably, the amplitude of product decreases as the length *L*_T_ of the dsDNA substrate increases, consistent with a dissociation step as shown in Scheme 1 (Figure 1). The individual uncorrected kinetic step size for different lengths of dsDNA varied from 14.3 to 16.4 as the length of dsDNA region decreased from 60 to 18 bp. Previous studies have shown that the last 8–10 bp of dsDNA can melt spontaneously in the presence of an active helicase[38]. Thus, it is essential to take into consideration the length of dsDNA that spontaneously melts when calculating the kinetic step size rather than using the full length of dsDNA. Hence, the value *L*_0_ used in Table 2 and equation 7 was set to 10 bp.

**Table 1:** Kinetic parameters of DNA unwinding by GST-nsp13.

*k_U_*(s^−1^)	*k*_d_ (s^−1^)	*k*_np_ (s^−1^)	*P*	m(bp) (*L_T_*)^a^	m (bp) (*L_0_*)^b^	m*k*_u_^a^(bps^−1^)	m*k*_u_^b^(bps^−1^)
30±2.0	4.5±0.3	1.4±0.3	0.8±0.03	14.3±3.2	9.3±2.0	429±28.6	279±18.6

**Table 2:** Individual kinetic step-size estimates (bp) for different lengths of dsDNA.

dsDNAlength	Number of steps (n)	Step-size (bp)^a^	Corrected step-size (bp)^b^
18	1.1	16.4	7.3
30	1.9	15.8	10.5
40	2.6	15.4	11.5
60	6.3	9.5	7.9

The kinetic parameters presented in this table are determined from the reaction mechanism presented in Figure 4A. Kinetic parameters are either ^a^uncorrected or ^b^corrected as described previously (48) by assuming that the last 10 base-pairs are unwound without direct helicase action. *P* is the processivity of DNA unwinding, *L_T_* is the total length of dsDNA and *L_0_* is the minimal length of dsDNA that is stable in the presence of active helicase and with last 10 bases unpaired. The kinetic step size *m* is defined as the (*L_T_*−*L_0_*)/*n*, where *n* is the number of intermediates.

After this adjustment, the corrected estimates of the kinetic step size ranged between 7.3 to 11.5 bp. Hence, the average kinetic step size *m*, was estimated to be 9.4±2.1 bp per step (Table 1).

Processivity is the probability that a helicase will move forward along the nucleic acid without dissociating during the unwinding reaction [38], [48]. Results in Figure 4B illustrate that as the length of the duplex increases, the amplitude of the respective unwinding time-course experiments decreases. The processivity of nsp13 was determined by plotting the amplitude against the number of steps with increasing base-pairs in the double-stranded region. The data were fit to equation *A* = *P^n^*. GST-nsp13 unwound nucleic acid with a processivity of 0.80±0.03 (Figure 4C).

### The Helicase Activity of nsp13 is enhanced by SARS-CoV Polymerase (nsp12)

**Figure 5:**
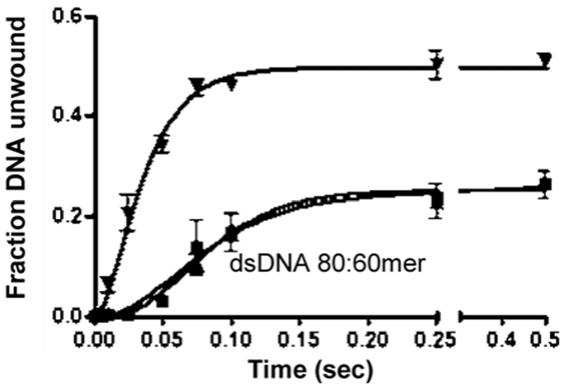
Effect of nsp12 on the unwinding activity of nsp13. Time course of DNA (5 nM. 80/60-mer) unwinding by 100 nM GST-nsp13 in the presence of 0 nM nsp12H_6_ (▴), 250 nM nsp12H_6_ (▪) and 500 nM nsp12H_6_ (▾). The products were separated and analyzed by 6% non-denaturing PAGE and the fraction of DNA unwound was plotted against reaction time. The experimental data were fit globally to equation (1) (Materials and Methods-Analyses of DNA unwinding) derived from Scheme 1 (Fig. 4A).

**Figure 6:**
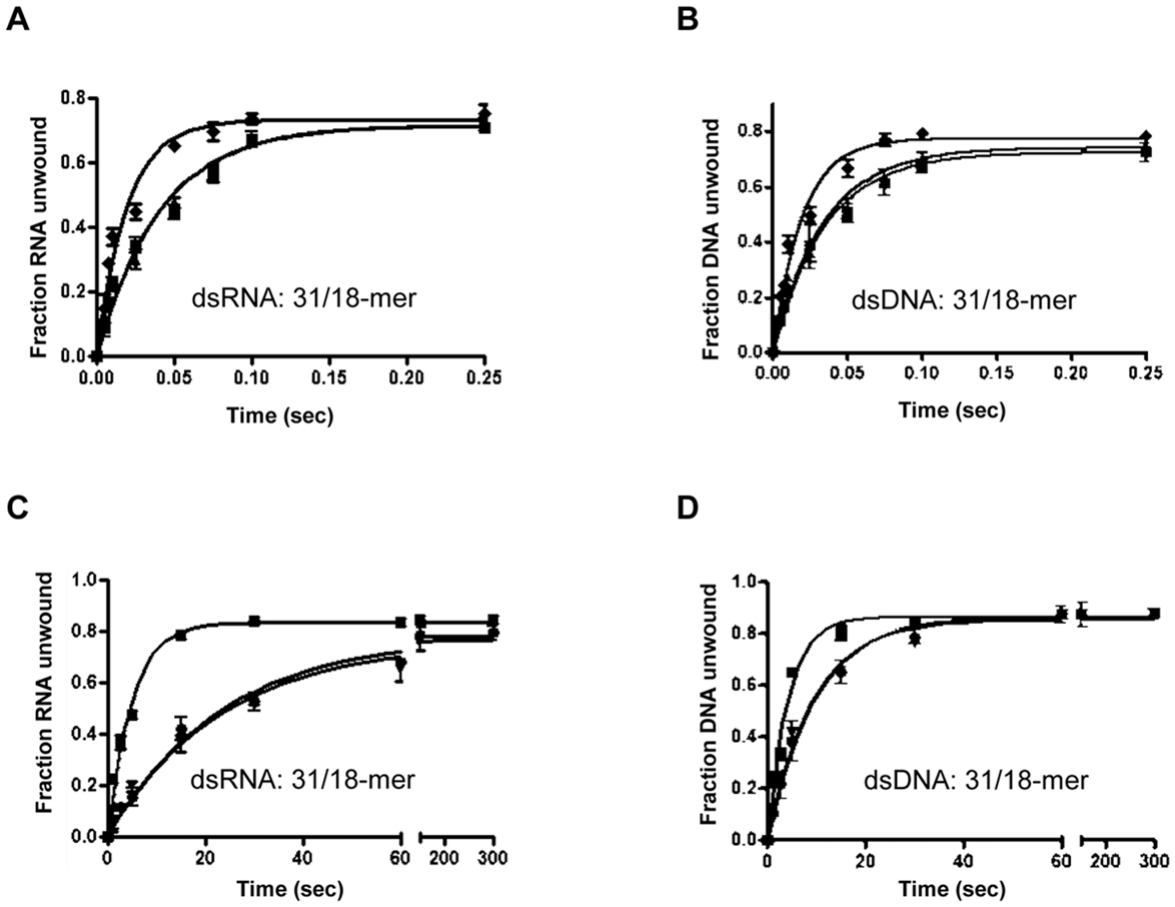
Comparison of the effect of nsp12 on nsp13 activity using RNA and DNA substrates. (A, B): Time course of (A) RNA (31/18-mer) and (B) DNA (31/18-mer) unwinding by GST-nsp13 alone (▪), or by GST-nsp13 with nsp12 (♦) or GST-nsp13 with Foot-and-Mouth Disease Virus RNA polymerase (▴). (C, D) Time course of (C) RNA (31/18-mer) and (D) DNA (31/18-mer) unwinding by H_6_-nsp13 alone (•), or by H_6_-nsp13 with nsp12 (▪) or by H_6_-nsp13 with Glutathione-S-transferase protein (without the nsp13 part) (▾).

Previous reports have shown that several DNA and RNA helicases interact with their corresponding polymerases. These interactions generally enhance the unwinding activity of helicases [49], [50], [51]. Evidence from yeast-two-hybrid experiments suggested that SARS-CoV polymerase (nsp12) interacts with nsp13 [31]. To investigate if nsp12 affects the helicase activity of nsp13 we monitored the unwinding of the 80/60-mer DNA substrates (5 nM) by GST-nsp13 (100 nM) in the presence of nsp12 (0 nM, 250 nM, and 500 nM) under conditions described in ‘Materials and Methods’. The results showed that 500 nM nsp12 clearly enhanced the unwinding rate (*mk_U_*, base-pairs per second) of nsp13 by ∼2-fold (Figure 5 and Table 3). This enhancement is accompanied by a change in the step size from 7.9 to 14.3 for this specific substrate (Table 3) demonstrating that the presence of nsp12 can change the rate and the mechanism of helicase activity. 500 nM of nsp12 was used because optimal unwinding of nucleic acid by nsp13 in the presence of nsp12 was observed at this concentration (Figure S3). Also, the effect of nsp12 on the ATPase activity of nsp13 was examined, and no enhancement of the ATPase activity was observed (data not shown).Our data suggest that this enhancement is independent of the type of substrate (dsDNA *vs.* dsRNA) or protein tag (GST *vs.* H_6_). A similar effect was observed when H_6_-nsp13 helicase activity was monitored in the presence of nsp12. The rate of unwinding the shorter 31/18-mer RNA substrate increased from 0.3±0.02 to 1.5±0.15 (*mk_U_*, base-pairs per second) and the rate of unwinding 31/18-mer DNA increased from 0.7±0.05 to 1.8±0.07 (*mk_U_*, base-pairs per second) (Figures 6C,6D and Table 4) with minimal change in the amplitude. However, the effect on the amplitude is more significant with the substrate with longer duplex region, 80/60-mer DNA substrate (Figure 5). Using the same 31/18-mer substrates, GST-nsp13 exhibited similar helicase activity enhancement as H_6_-nsp13 (Figures 6A, 6B, and Table 4). Notably, this enhancement is specific, as it was not observed when the nsp12 SARS RdRp was substituted with the foot-and-mouth disease virus RdRp (FMDV 3Dpol) (Figures 6A, 6B, and Table 4).

**Table 3:** Kinetic parameters for the effect of nsp12 on the unwinding activity of GST-nsp13.

Enzyme	Amplitude	*k_U_* (steps/s)	Number of steps	m (bp/step) *(L_T_,uncorrected)^a^*	m(bp/step) *(L_0_,corrected)^b^*	m*k_u_* ^b^(bps^−1^)
nsp13	0.25±0.02	29.3±0.1	6.3	9.5	7.9	231.5±0.6
nsp12+nsp13	0.52±0.01	37.6±0.0	3.5	17.1	14.3	538.0±0.1

Total length of dsDNA (60 bp) at the outset of reaction ^(a)^.

Final 10 bps are separated without direct helicase action ^(b)^.

Kinetic parameters were determined by nonlinear least-squares analysis of data from Figure 5. Experiments were performed three times.

**Table 4:** Comparison of the effect of nsp12 on the unwinding parameters of GST-nsp13 and H_6_-nsp13 on RNA and DNA substrates (31:18-mer).

Enzyme	Substrate	Amplitude	*k_U_*(s^−1^)	*m* (bp) (*L_0_*, *corrected*)^b^	m*k*_u_ b(bps^−1^)
GST-nsp13	RNA	0.72±0.02	24.15±2.1	7.3	176±15
GST-nsp13+nsp12	RNA	0.73±0.02	51.06±4.7	7.3	373±34
GST-nsp13+FMDV pol	RNA	0.72±0.02	24.54±2.0	7.3	179±15
GST-nsp13	DNA	0.73±0.02	28.41±2.2	7.3	207±16
GST-nsp13+nsp12	DNA	0.78±0.02	50.89±3.9	7.3	371±29
GST-nsp13+FMDV pol	DNA	0.74±0.02	29.30±2.9	7.3	214±21
H_6_-nsp13	RNA	0.78±0.01	0.04±0.003	7.3	0.3±0.02
H_6_-nsp13+nsp12	RNA	0.83±0.02	0.20±0.02	7.3	1.5±0.15
H_6_-nsp13+GST	RNA	0.76±0.02	0.04±0.003	7.3	0.3±0.02
H_6_-nsp13	DNA	0.87±0.01	0.10±0.007	7.3	0.7±0.05
H_6_-nsp13+nsp12	DNA	0.87±0.01	0.24±0.01	7.3	1.8±0.07
H_6_-nsp13+GST	DNA	0.86±0.01	0.11±0.006	7.3	0.8±0.04

b Correction for bp separated without direct helicase action as described in Table 1 and reference 48.

Kinetic parameters were determined by nonlinear least-squares analysis of data from Figure 6. Experiments were performed three times.

To confirm whether purified nsp12 and nsp13could physically interact in the absence and presence of nucleic acid, we performed a GST pulled down and gel mobility shift assays respectively. The results show that the GST-nsp13 and nsp12-H_6_ could physically interact in the presence and absence of nucleic acid (Fig. S4A and S4B)

## Discussion

Our long-term goal is to understand the mechanism of SARS-CoV replication and the specific contribution of nsp13, as a prerequisite for the development of antivirals. This study focused on the biochemical mechanism of SARS-CoV nsp13 and how it is affected by nsp12. Our results provide the first mechanistic glimpses into the functional interactions between key components of the SARS-CoV replication complex (RC) and allow comparisons with other RCs.

Sequence alignment of nsp13 with other helicases suggests that the SARS-CoV nsp13 belongs to the SF1 family of helicases [34]. However, several biochemical aspects of the enzymatic mechanism of SARS-CoV nsp13 are still unknown, including ATP hydrolysis, translocation along the nucleic acid, and the unwinding rate. The present study fills this knowledge gap and establishes the minimal kinetic mechanism of the enzyme. Moreover, it provides the first insights into how interactions between the helicase and polymerase enzymes enhance the efficiency of replication in coronaviruses.

Previously published work with nsp13 fused to hexahistidine or MBP tags and expressed in bacterial systems demonstrated that the helicase activity of these enzymes was considerably lower compared to other viral, bacterial, or eukaryotic helicases [34], [35]. To determine whether nsp13 is indeed a ‘slow’ enzyme, we prepared a GST-nsp13 construct and expressed it in a bacterial and baculovirus expression systems. GST-nsp13 protein expressed in bacterial systems was insoluble, whereas when expressed in a baculovirus expression system it was highly soluble (Figure S2). GST-nsp13 expressed in baculovirus displayed much higher unwinding and ATPase activities than the H_6_- or MBP-tagged proteins (Figure 1 and Figure 2). This enzyme showed same polarity as reported before for two other variants of nsp13 [34], [35]. GST-nsp13 requires at least a five nucleotide 5′-end single strand overhang for efficient unwinding similar to human coronavirus 229E helicase [52]. GST-nsp13 can unwind secondary structures of nucleic acid (such as those present in viral genome) as long as a sufficient 5′ single strand overhang is available. Unlike MBP-nsp13 and H_6_-nsp13, GST-nsp13 has a helicase activity comparable to other viral, bacterial and eukaryotic helicases. We and others have been unable to hydrolytically remove the affinity tags from nsp13 [34], [35] presumably because the engineered cleavage site is not easily accessible to the protease. Notably, exogenously added GST did not have any effect on the rate of nucleic acid unwinding by both H_6_-nsp13 (Figures 6C and 6D) and MBP-nsp13 (data not shown), suggesting that the higher activity of GST-nsp13 is not a result of a GST artifact. It is not clear why GST-nsp13 is more active than H_6_-nsp13. The two proteins appear to have comparable affinities for dsDNA as seen in gel shift experiments (results not shown). Comparison of the ATP hydrolysis rate by GST-nsp13 and H_6_-nsp13 showed that H_6_-nsp13 hydrolyzes ATP slower than GST-nsp13. Hence, based on these experiments we decided to biochemically characterize the GST-nsp13 enzyme, although most of the experiments were also carried out with H_6_-nsp13.

Our detailed biochemical analysis showed that GST-nsp13 unwinds DNA at a rate (*k*_u_) of 30 steps per second, with each step being approximately 9.3 bps. The kinetic characterization of several helicases have shown that the step size for DNA unwinding varies between (1–20 bps) [53], [54], [55], [56]. We used Scientist 3.0 (Micromath) software for obtaining the kinetic parameters, and these parameters were also confirmed with the Dynafit 4.0 (BioKin Ltd, MA) and Kintec Explorer (Kintek Corporation, PA) software packages. Helicase processivity provides a measure of the fraction of unwound nucleic acid before dissociation.

Helicase processivity provides a measure of the fraction of unwound nucleic acid before dissociation. Based on our data (Figure 4C) the estimated value of nsp13 processivity is 0.8, which is lower than that reported for the very processive RecBCD and UVrD DNA helicases and higher than that of DdA helicase [37].

Our data suggest that nsp12 and nsp13 are in contact during viral replication, and are consistent with previous yeast two hybrid system studies, which have shown that these two proteins can physically interact with each other [31]. The enhancement of nsp13 activity in the presence of nsp12 is specific as it does not occur in the presence of another RdRp, such as FMDV 3Dpol. Moreover, it was observed not only for GST-nsp13, but also for the H_6_-nsp13 (Figures 6C and 6D) and MBP-nsp13 (data not shown), demonstrating that it is not an artifact of the fusion tag. To our knowledge, this is the first report of a nidovirus RNA helicase activity enhancement by an RdRp. The synergy between the two enzymes is likely to be important for coronavirus replication, as is the case in other bacterial, mitochondrial, bacteriophage DNA- or viral RNA- replication systems. In these cases, the DNA polymerase [49], [51], [57], [58], [59] or the RNA polymerase [60] were reported to enhance unwinding by the corresponding helicase.

Interestingly, nsp12 translocates on the template strand in a 3′→5′ direction during RNA synthesis. On the other hand, unlike pestivirus or flavivirus helicases, SARS-CoV nsp13 binds a 5′ overhang and moves 5′→3′ to unwind double stranded RNA [34], [35]. Despite the fact that the enzymatic functions of nsp13 and nsp12 have opposite polarities (5′-3′ vs 3′-5′ of the template strand)nsp12 and nsp13 may work together to carry out the following RC functions: 1) Synthesis of subgenomic transcripts containing the leader sequence derived from the 5′end of the genome [34], [35]. To accomplish this, nsp12 would have to copy RNA in a 3′→5′ template direction, followed by pausing at transcription regulatory element sites. Such pauses may trigger motion in the opposite direction allowing nsp13 (aided by nsp12 and possibly other viral and host proteins) to unwind the 3′ end of the nascent RNA primer and facilitate transfer to the complementary region of the 5′-leader genomic sequence [34], [35]. 2) Data in Figure 3B suggest that nsp13 is capable of disrupting secondary structures of nucleic acid, only when they can be accessed by an upstream 5′-single stranded region. Hence, stem-loop “roadblocks” are likely to be cleared by RCs that approach the stem loop from the 5′ ssRNA direction. Ongoing biochemical experiments focus on characterizing RC functions that involve nsp13.

In conclusion, we have established a minimal kinetic mechanism for the SARS-CoV helicase and have demonstrated that the unwinding efficiency of nsp13 is enhanced by the SARS-CoV RdRp.

## Materials and Methods

### Cloning, Expression and Purification of GST-nsp13 helicase

The coding region of nsp13 was amplified by PCR using 5′*BamHI*-nsp13-ATGCTAGGATCCGCTGTAGGTGCTTGTG-3′ as the forward primer and 3′*XhoI*-nsp13-GCTGACCTCGAGTTATTGTAATGTAGCCACATTGC3′ as the reverse primer. The PCR amplicons were digested with *BamH I* and *XhoI* followed by ligation into expression vector pGEX-4T-1 (Amersham Biosciences). After confirming the amplicon nucleotide sequences we used the flanking *RsrII* and *XhoI* restriction sites to subclone GST-nsp13 into the pFASTBAC1 vector (Invitrogen). In the final pFASTBAC1-GST-nsp13 construct, GST was fused at the N-terminus of nsp13. This plasmid was transformed into *E. coli* DH10Bac cells for transposition of GST-nsp13 from pFASTBAC1 into a bacmid, which is a baculovirus shuttle vector with a baculovirus-specific promoter (*i.e*, the polyhedrin or p10 promoter). The bacmid was propagated in *E. coli* DH10Bac as a large plasmid that confers resistance to kanamycin and can complement a *lac*Z deletion present on the chromosome to form blue (Lac+) colonies in the presence of a chromogenic substrate such as Blue-gal or X-gal and the inducer IPTG. Insertion of GST-nsp13 into the bacmid disrupted expression of *lac*Zα, allowing the selection of GST-nsp13-containing bacmids (white colonies) and isolation of DNA using the High Purelink DNA isolation kit (Invitrogen). Following confirmation of GST-nsp13 gene insertion in the bacmid, the recombinant bacmid was then transfected into HighFive Insect cells using cellfectin transfection reagent (Invitrogen). After 72 hrs we harvested supernatants containing the 1^st^ recombinant baculovirus stock (P1). In order to amplify the viral stock, the harvested P1 was used to infect fresh HighFive Insect cells at an MOI of 0.1 and the supernatant was collected 48 hrs post-infection. This resulted in 100-fold amplification of the virus (P2). The P2 stock was subsequently used to infect fresh monolayer cultured HighFive insect cells for GST-nsp13 expression. After 48 hrs, cells were harvested and resuspended in the “Binding Buffer” (137 mM NaCl, 1.94 mM K_3_PO_4_, 8.06 mM Na_3_PO_4_, and 2.7 mM KCl, pH 7.4; phosphate buffered saline). The cells were lysed by sonication and the lysate was passed through Glutathione Sepharose beads (Amersham Biosciences). The beads were washed with 20 column volumes of Binding Buffer followed by elution of GST-nsp13 using 10 mM reduced glutathione in 50 mM Tris, pH 8.0. Elution fractions were analyzed by SDS–PAGE and purified GST-nsp13 was visualized by Coomassie-Brilliant Blue staining as 92-kDa protein product, in line with the expected mass of the fusion protein. The protein was further purified by size exclusion chromatography on a Superdex 200 10/300GL column (GE Healthcare). The purified protein (∼95% pure) was stored at −80°C in a buffer containing 50 mM Tris-HCl, pH 8.0, 200 mM NaCl, 1 mM dithiothreitol (DTT) and 5% glycerol. Despite repeated attempts under several conditions, the GST N–terminal tag could not be cleaved using thrombin.

### MBP-nsp13 Cloning, Expression, and Purification

nsp13 was cloned into the pMAL-p4x using *BamHI* and *XbaI* at the 5′ and 3′ ends. The resulting pMal-nsp13 was used to transform *E. coli* TB1 cells (New England Biolabs). MBP-nsp13 was expressed and purified by amylose affinity chromatography (New England Biolabs). MBP-nsp13 was further purified by size-exclusion chromatography on a Superdex-200 10/300GL column (GE Healthcare) run under isocratic conditions with 20 mM Tris-HCl, pH 7.5, 200 mM NaCl, 1 mM DTT, 0.1 mM EDTA, and 5% glycerol. Fractions containing pure nsp13 were concentrated and stored at −80°C.

### H_6_-nsp13 Cloning, Expression and Purification

Hexahistidine containing nsp13 was PCR amplified using the same primers mentioned above with *BamHI* and *SalI* restriction sites. The PCR amplicon was digested with *BamHI* and *SacI*, and ligated into pET-28a. A similar version of the plasmid was also obtained from Dr. John Ziebuhr (Justus Liebig University Giessen, Germany). Protein expression and purification was performed as described previously using Talon beads [35]. The protein was eluted with 25 mM Hepes pH 7.0, 0.5 M NaCl, 200 mM imidazole, 0.1% Triton X-100. It was further purified on a Superdex75 10/300GL column (20 mM Tris-HCl, pH 6.8, 200 mM NaCl, 1 mM DTT, and 5% glycerol. Fractions containing the desired protein were concentrated and stored at −80°C.

### nsp12-H_6_ Cloning, Expression, and Purification

C-terminal hexahistidine containing nsp12- (nsp12-H_6_) was cloned, expressed and purified as described previously [61], [62]. Briefly nsp12 was PCR amplified using 5′*SacII* nsp12-GCGGGTACCCC GCGGTGGATCTGCGGATGCATCAA-3′ and 5′*BamHI* nsp13-ATGCTAGGATCCGCTGTAGG TGCTTGTG (forward primer), and 5′*BamHI* nsp13-GCGCGATCGGGATCCCTGCAAGACT GTATGT (reverse primer). The PCR amplicon was digested with *SacII* and *BamHI*, and ligated into expression vector pET26-Ub-CHis_6_ followed by subcloning of the ubiquitin-nsp12-His_6_ fusion gene (Ub-nsp12-CHis_6_) into vector pASK3 (IBA). Protein expression and purification was performed as described previously using Talon beads [35]. The protein was eluted with 20 mM HEPES pH 7.4, 0.5 M NaCl, 200 mM imidazole, 0.1% Triton X-100 and further purified on a Superdex75 10/300GL column (20 mM HEPES pH 7.4, 100 mM NaCl, 1 mM DTT, 0.1% Triton-X 100, and 5% glycerol). Fractions containing the desired protein were concentrated and stored at −80°C.

### Expression and Purification of FMDV 3Dpol

Expression and purification were as previously described [63]. Briefly, pET-28a-FMDV 3Dpol plasmid was transformed into the Rosetta 2 expression strain (Novagen). Kanamycin-resistant colonies were grown at 37°C and protein expression was induced at A_600_ of 0.9–1.0 by the addition of 1 mM isopropyl b-D-1-thiogalactopyranoside (IPTG) and the cells were allowed to grow for 3 more hrs after induction. The cells were harvested by centrifugation (4,500 g, 20 min) and stored at −20°C. Frozen cell pellets were resuspended in buffer A (25 mM Tris-HCl pH 8.0, 500 mM NaCl and 5% glycerol). The protein was purified by nickel-affinity chromatography with a gradient of 25 mM to 500 mM imidazole in buffer A. Fractions containing pure protein (,95%) were pooled and dialyzed against the storage buffer containing 12.5 mM Tris-HCl pH 8.0, 100 mM NaCl and 50% glycerol.

### GST Pull Down of Enzymes

Purified GST nsp13, nsp12H_6_ and FMDV 3D pol were treated with DNase I (Fermentas), RNAse A/T1 mix (Fermentas), followed by dialysis against (137 mM NaCl, 1.94 mM K_3_PO_4_, 8.06 mM Na_3_PO_4_, and 2.7 mM KCl, pH 7.4; phosphate buffered saline). GST-nsp13 was incubated with nsp12H_6_ or FMDV 3D pol at 4°C for 2 hrs, followed by incubating the mixture with 50% slurry of glutathione-conjugated Sepharose beads (Amersham Biosciences), and the binding reaction was further incubated for 1 hr at 4°C. Precipitates were washed extensively with phosphate buffer saliine. Proteins bound to glutathione beads were eluted using a buffer containg 10 mM Reduced Gluthatione (Sigma Aldrich) and 50 mM Tris pH 8.0 and separated on a SDS–PAGE and purified proteins were visualized by Coomassie-Brilliant Blue staining.

### Gel Mobility Shift Assay

To determine if GST-nsp13could form a complex with nsp12-H_6_ in the presence of nucleic acid substrate, we performed gel mobility shift assay. We measured the binding of 60/40-mer (20ss:40ds) DNA substrates (Fig. S1), at specific concentrations of GST-nsp13 and varying concentrations of nsp12H_6_ in a reaction mixture containing 20 mM HEPES pH 7.5, 20 mM NaCl, 5 mM MgCl_2_, 1 mM DTT, 0.1 mg/ml BSA and 5% glycerol at 30°C for 10 minutes. The concentration for the 5′-Cy3-labeled dsDNA substrates was 5 nM. Samples were electrophoresed at 100 V for 1.5 h at 4°C on a 5% non-denaturing polyacrylamide gel, using 89 mM Tris borate pH 8.2. Gels were scanned in a PhosphorImager (FLA 5000, FujiFilm).

### Nucleic Acid Substrates

Synthetic oligonucleotides were purchased from Integrated DNA Technologies (Coralville, IA). Sequences of the DNA and/or RNA substrates are shown in Supplementary Fig. 1. Concentrations were determined spectrophotometrically using absorption at 260 nm and their extinction coefficients. Unlabeled oligonucleotides were annealed to corresponding 5′-Cy3 labeled 18-mer, 30-mer, 40-mer and 60-mers in 50 mM Tris pH 8.0, 50 mM NaCl at a ratio of 1∶1.2 by heating at 95°C for 5 min and cooling slowly to room temperature. Unlabeled 18-mer, 30-mer, 40-mer and 60-mers were used as traps for the helicase assay.

### Initial Characterization of Helicase Activity

Helicase activity was measured by incubating 100 nM GST-nsp13 with 5 nM 60/40-mer DNA substrate (Figure S1) in a reaction buffer containing 20 mM HEPES pH 7.5, 20 mM NaCl, 1 mM DTT, 0.1 mg/ml BSA, 5 mM MgCl_2_, and 2 mM ATP at 30°C for various times [34]. The reaction mixture also contained 1 µM unlabeled 40-mer DNA as trap. Reactions were quenched by the addition of equal volume of loading buffer (100 mM EDTA, 0.2% SDS and 20% glycerol). Unless otherwise mentioned, reactant concentrations refer to the final concentration in the reaction mixture. The released single-stranded DNA (ssDNA) product and unwound double-stranded DNA (dsDNA) were resolved on a 6% non-denaturing-PAGE (polyacrylamide gel electrophoresis) using a running buffer containing 89 mM Tris-Borate pH 8.2, and run for 2 hours at 4°C and 150 V. The controls for measuring maximum unwinding were dsDNA denatured by heating for 5 min at 95°C and loading immediately on the gel as suggested by Ahnert et al. [57]. In this and subsequent assays the gels were scanned with a phosphorimager (FLA 5000, FujiFilm). The band intensities representing ssDNA and dsDNA were quantitated using the ImageQuant software (Pharmacia). The fraction of unwound DNA was plotted against time and the kinetic parameters described in the mechanism were determined by non-linear regression using the Graphpad Prism (GraphPad Inc.) and Scientist (Micromath) programs.

### Pre-Steady State Kinetic Assay

The assay conditions for single turnover helicase activity measurements were similar to those used for initial characterization except that the concentration of nsp13 was in excess over the DNA substrates. The reactions were carried out in 20 mM HEPES (pH 7.5), 20 mM NaCl, 5 mM MgCl_2_, 1 mM DTT, 0.1 mg/ml BSA, and 5% glycerol at 30°C using a Rapid Chemical Quench Flow instrument (KinTek Corp.) [43], [57]. GST-nsp13 (100 nM) and DNA substrates (5 nM) were loaded into one of the sample loops (15 µl), whereas ATP (2 mM), and unlabeled DNA substrate (1 µM) were loaded into the other sample loop (15 µl). Samples were rapidly mixed and the reaction was quenched with 100 mM EDTA, 0.2% SDS, and 20% glycerol after desired time intervals (5 ms to 1 s). Reaction products were resolved and quantitated as described above. Experiments were performed at least three times. To determine the effect of nsp12 (SARS-CoV RdRp) on the helicase activity of nsp13, the experiment was as described above except that varying concentrations of nsp12-H_6_, GST-nsp13 (100 nM) and 5′-Cy3-labeled 80/60-mer (20ss:60ds) DNA (5 nM) were loaded together into one of the sample loops.

### Analyses of DNA Unwinding

To obtain the kinetic parameters associated with DNA unwinding by nsp13, the fraction of unwound DNA was plotted against time. Data fitting was carried out by non-linear regression using Dynafit 4.0 (BioKin Ltd, MA) Scientist 3.0 (Micromath), Prism 5.0 (GraphPad Inc.), and Kintec Explorer (Kintek Corporation, PA) software packages as described below. The fraction of ssDNA molecules formed after time ‘t’, *f_ss_(t)* for an n-step unwinding mechanism [41] depicted in Fig. 1 is defined as:
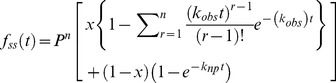
(1)Where *k*_obs_ is the sum of the unwinding rate and dissociation constants *k*_u_ and *k*_d_:
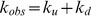
(2)
*k*_np_ is a macroscopic rate constant for conversion of nonproductive (E-DNA)_np_ to productive Enzyme-DNA complexes (E-DNA)_L_

The processivity in equation 1 is defined as
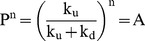
(3)The terms m and N are step size and average number of unwound base-pairs, and A is the amplitude of unwinding reaction. The parameter *k*_np_ is a macroscopic constant associated with the conversion of non-productive to productive nsp13-DNA complex; ‘x’ is the fraction of DNA molecules bound to helicase in complexes that are productive for DNA unwinding and is defined as:
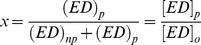
(4)Where [*ED*]_p_ is productive enzyme-DNA complex and [*ED*]_o_ is the sum of productive [*ED*]_p_ and non-productive [*ED*]_np_ enzyme-DNA complex. Equation (1) can be solved by the method of Laplace transformation. The solution for this equation is:
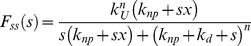
(5)where s is the Laplace variable of the fraction of ssDNA product formed over time, f_ss_(t). The inverse Laplace transform, L^−1^, can be estimated by numerical integration capabilities of the software Scientist to obtain *f_ss_*(t) as shown in equation 6:
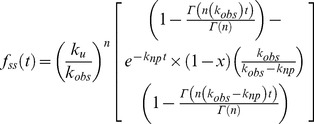
(6)This mechanism assumes that nsp13 unwinds total DNA in n number of steps of equal m-step size.
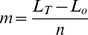
(7)where *L_T_* equals the total length of dsDNA in bp, and *L_0_* equals the minimal length of dsDNA that is stable in the presence of an active helicase. The resulting individual kinetic step-size estimates may then be used to obtain an average kinetic step-size for the helicase under investigation.

### ATP Hydrolysis Assay

ATP hydrolysis measurements were carried out under single turnover conditions similar to those used in unwinding assays. 100 nM GST-nsp13 and 5 nM DNA substrates were loaded into one sample loop (15 µl) and various concentrations of γ^-32^P-ATP (2, 5, 12, 25, or 50 µM), together with 1 µM unlabeled 18-mer DNA (to prevent re-annealing on the unwound labeled strand) were loaded into the other sample loop (15 µl). Both samples contained a reaction buffer consisting of 20 mM HEPES pH 7.5, 20 mM NaCl, 5 mM MgCl_2_, 1 mM DTT, 0.1 mg/ml BSA, and 5% glycerol. Samples were rapidly mixed at 30°C and reactions were allowed to proceed for desired time periods ranging between 0.005 to 0.5 s for GST-nsp13 or 0.005 to 5 s for H_6_-nsp13 and MBP-nsp13 prior to quenching with 100 mM EDTA, 0.2% SDS, and 20% glycerol. Reaction products were separated by thin-layer chromatography on polyethyleneimine-cellulose F plates (Merck) using 0.5 M LiCl as the liquid phase and visualized by autoradiography. The monophosphate (Pi) hydrolysis product was quantitated using ImageQuant (Amersham) and used to calculate hydrolyzed fraction of ATP. Experiments were performed twice and kinetic parameters were determined using the Prism (GraphPadInc) software.

## Supporting Information

Figure S1 **Oligonucleotides and substrates used in this study.** The Cy3-labeled strands are marked by asterisks. The sequences in red are self-annealing within the longer strand, while the green sequences denote the complementary sequences in the two strands.
(TIF)

Figure S2 **Expression and Purification of GST-nsp13.** *Left panel* (first four lanes): SDS-PAGE gel of GST-nsp13 expressed in *E.coli* BL21 cells. All GST-nsp13 is at the pellet and none is in the combined and concentrated elution fraction). *Right panel* (last four lanes): SDS-PAGE gel of GST-nsp13 expressed in baculovirus expression system and purified as described in ‘Materials and Methods’.
(TIF)

Figure S3 **Effect of nsp12-H_6_ on the unwinding activity of GST-nsp13.** The helicase activity of GST-nsp13 (100 nM) was assessed on 5 nM 60/40-mer (20ss:40ds) as DNA substrate in the presence of varying concentrations of nsp12-H_6_ (50–500 nM) at 30°C for 0.1 sec. The products were separated on a non-denaturing 6% polyacrylamide gel and visualized as described under ‘Materials and Methods’.
(TIF)

Figure S4 **Interaction of GST-nsp13 and nsp12H_6_.** A) Purified GST nsp13, nsp12H_6_ and FMDV 3D pol were dialyzed against (137 mM NaCl, 1.94 mM K_3_PO_4_, 8.06 mM Na_3_PO_4_, and 2.7 mM KCl, pH 7.4; phosphate buffered saline). GST-nsp13 was incubated with nsp12H_6_ or FMDV 3D pol at 4°C for 12 hrs, followed by incubating the mixture with 50% slurry of glutathione-conjugated Sepharose beads (Amersham Biosciences), and the binding reaction was further incubated for 4 hrs at 4°C. Precipitates were washed extensively with phosphate buffer saliine. Proteins bound to glutathione beads were eluted and separated on a SDS–PAGE and purified proteins were visualized by Coomassie-Brilliant Blue staining. The left and right panels represent the SDS-PAGE for GSTnsp13-nsp12 interaction and the GST-nsp13/FMDV 3D pol data respectively. B) Binding of 60/40-mer (20ss:40ds) DNA substrates, with GST-nsp13 and varying concentrations of nsp12H_6_ was assessed using a gel mobility shift assay. Samples were analysed on a 5% non-denaturing polyacrylamide gel.
(TIF)
