# Supplementary figures and images for: Mechanism of Nucleic Acid Unwinding by SARS-CoV Helicase
Source: PLoS One. 2012 May 15;7(5):e36521. doi: 10.1371/journal.pone.0036521 (PMC3352918; doi:10.1371/journal.pone.0036521)

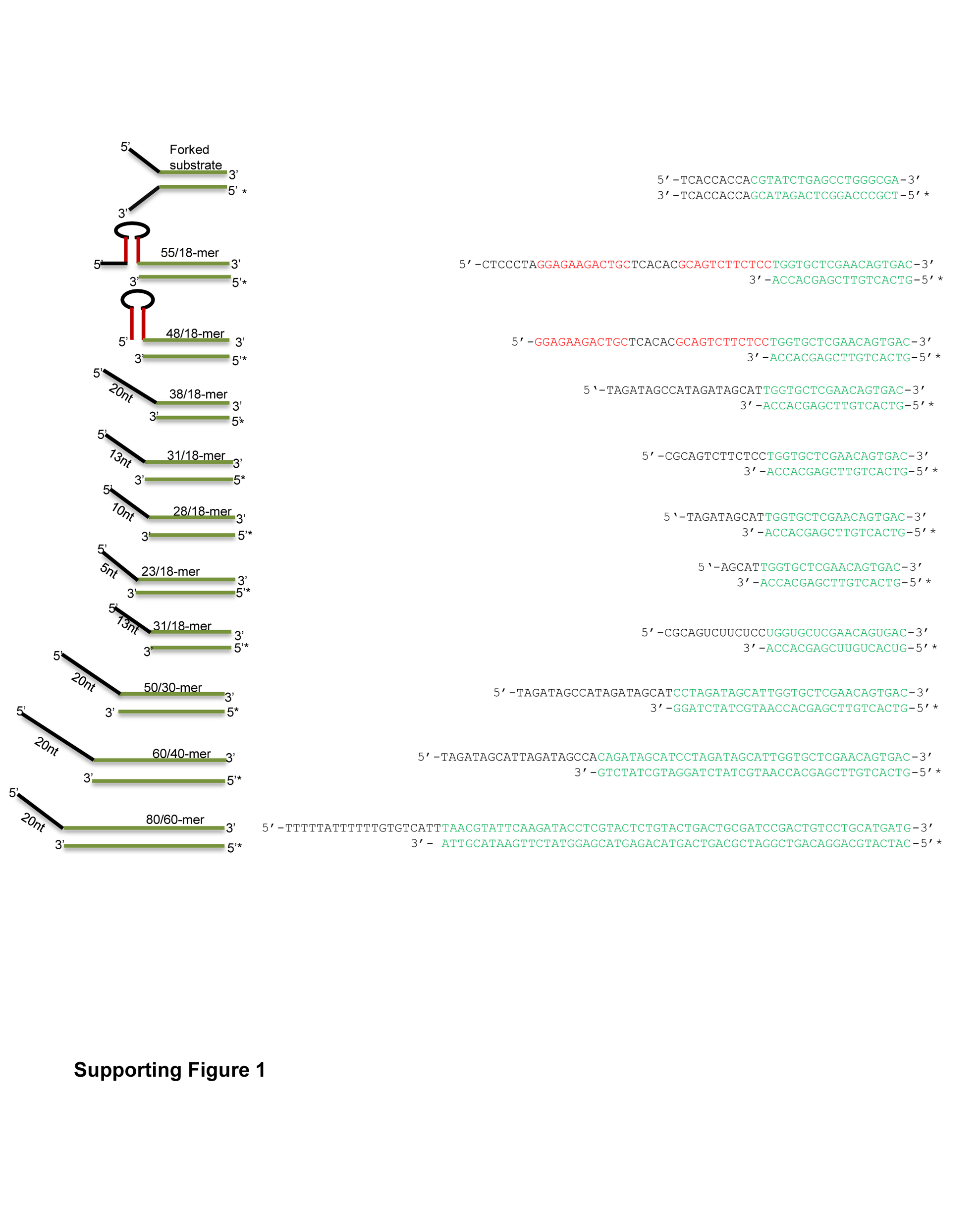

Supplement: Figure S1 — Oligonucleotides and substrates used in this study. The Cy3-labeled strands are marked by asterisks. The sequences in red are self-annealing within the longer strand, while the green sequences denote the complementary sequences in the two strands. (TIF) [file pone.0036521.s001.tif]

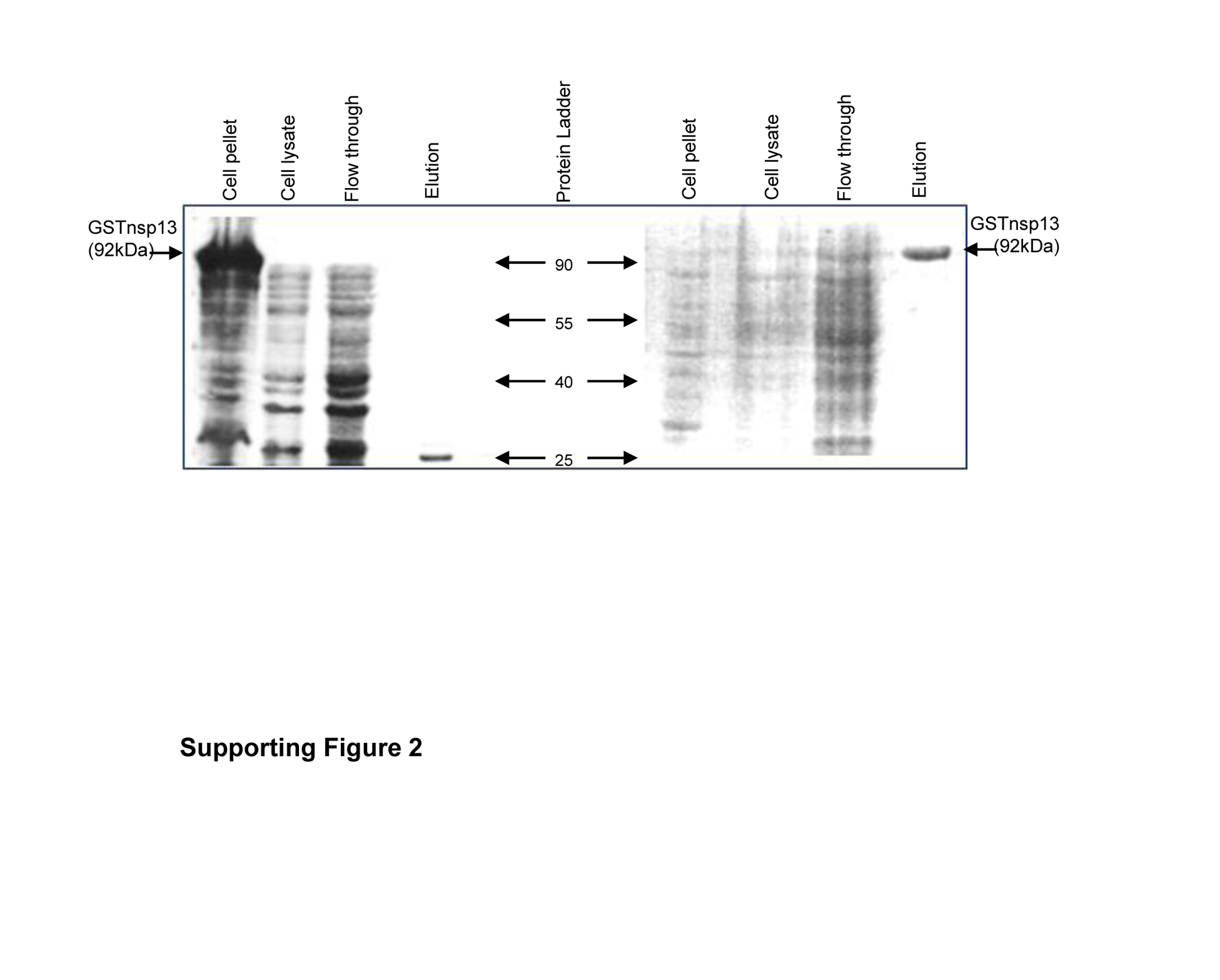

Supplement: Figure S2 — Expression and Purification of GST-nsp13. Left panel (first four lanes): SDS-PAGE gel of GST-nsp13 expressed in E.coli BL21 cells. All GST-nsp13 is at the pellet and none is in the combined and concentrated elution fraction). Right panel (last four lanes): SDS-PAGE gel of GST-nsp13 expressed in baculovirus expression system and purified as described in ‘Materials and Methods’. (TIF) [file pone.0036521.s002.tif]

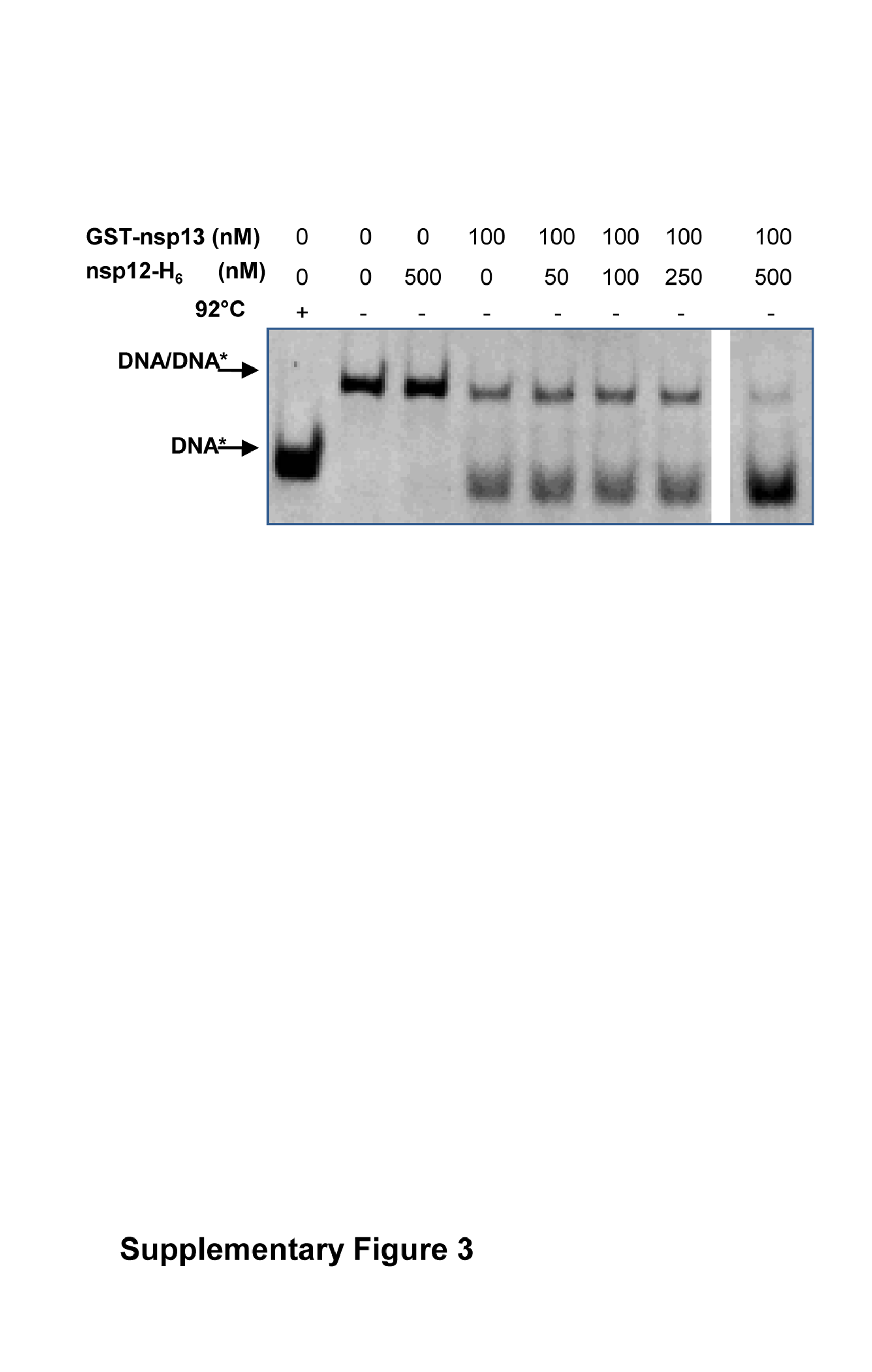

Supplement: Figure S3 — Effect of nsp12-H6 on the unwinding activity of GST-nsp13. The helicase activity of GST-nsp13 (100 nM) was assessed on 5 nM 60/40-mer (20ss:40ds) as DNA substrate in the presence of varying concentrations of nsp12-H6 (50–500 nM) at 30°C for 0.1 sec. The products were separated on a non-denaturing 6% polyacrylamide gel and visualized as described under ‘Materials and Methods’. (TIF) [file pone.0036521.s003.tif]

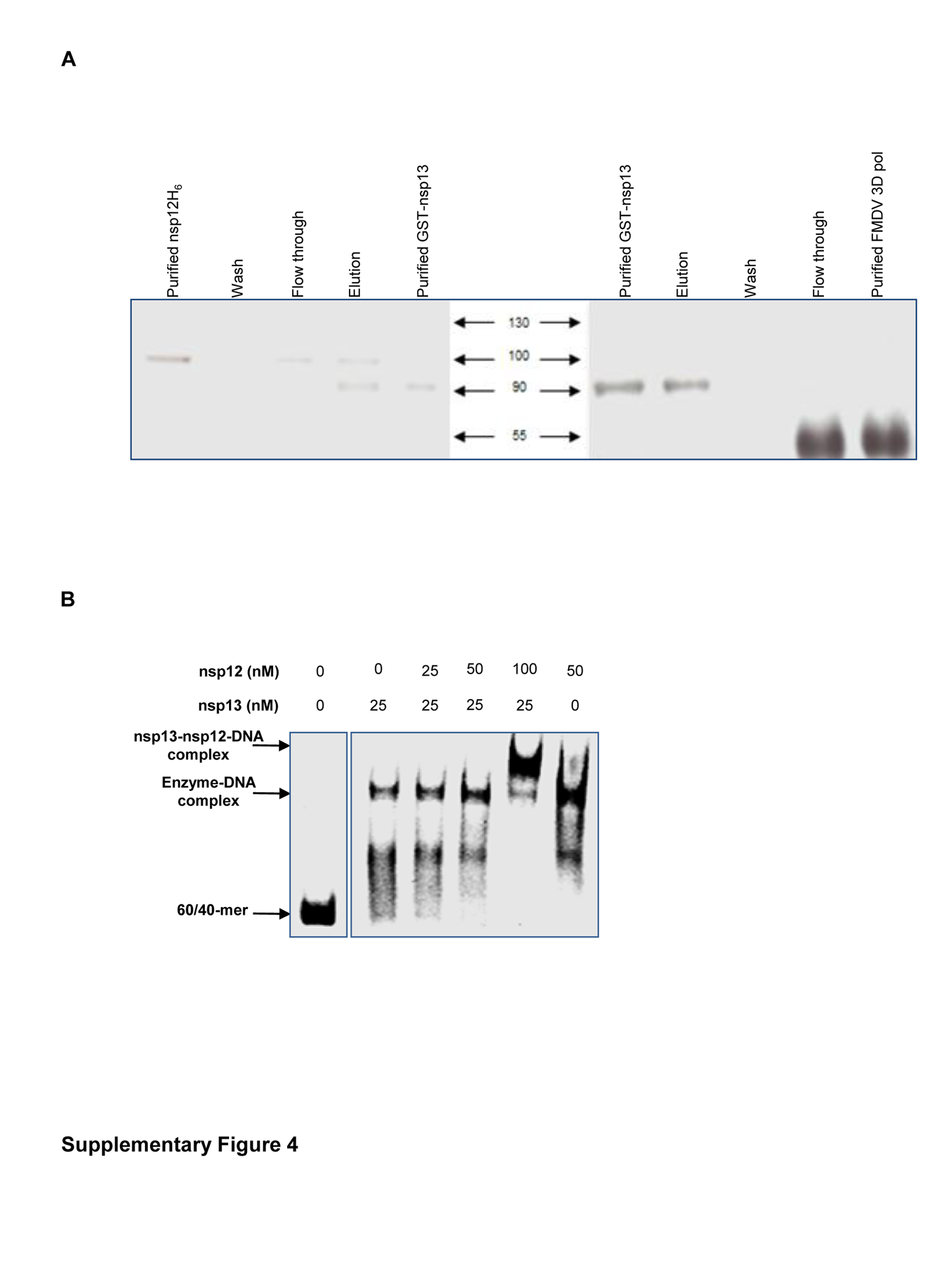

Supplement: Figure S4 — Interaction of GST-nsp13 and nsp12H6. A) Purified GST nsp13, nsp12H6 and FMDV 3D pol were dialyzed against (137 mM NaCl, 1.94 mM K3PO4, 8.06 mM Na3PO4, and 2.7 mM KCl, pH 7.4; phosphate buffered saline). GST-nsp13 was incubated with nsp12H6 or FMDV 3D pol at 4°C for 12 hrs, followed by incubating the mixture with 50% slurry of glutathione-conjugated Sepharose beads (Amersham Biosciences), and the binding reaction was further incubated for 4 hrs at 4°C. Precipitates were washed extensively with phosphate buffer saliine. Proteins bound to glutathione beads were eluted and separated on a SDS–PAGE and purified proteins were visualized by Coomassie-Brilliant Blue staining. The left and right panels represent the SDS-PAGE for GSTnsp13-nsp12 interaction and the GST-nsp13/FMDV 3D pol data respectively. B) Binding of 60/40-mer (20ss:40ds) DNA substrates, with GST-nsp13 and varying concentrations of nsp12H6 was assessed using a gel mobility shift assay. Samples were analysed on a 5% non-denaturing polyacrylamide gel. (TIF) [file pone.0036521.s004.tif]
